# Beyond immune surveillance: IFN-γ and PD-L1 as shapers of tumor plasticity in Non-Small Cell Lung Cancer (NSCLC)

**DOI:** 10.3389/fimmu.2026.1945729

**Published:** 2026-10-07

**Authors:** Stefania Angelicola, Mariateresa Frascino, Ilaria C. Chillico, Patrick L. S. Michel, Damiano Pistone, Maria Sofia Semprini, Olga Maria Pittino, Andrea Ardizzoni, Niccolò Leandro Alessio, Francesco Agustoni, Francesco Gelsomino, Pier-Luigi Lollini, Arianna Palladini

**Affiliations:** 1 IRCCS Azienda Ospedaliero-Universitaria di Bologna, Bologna, Italy; 2 Department of Molecular Medicine, University of Pavia, Pavia, Italy; 3 Unit of Radiology, Fondazione IRCCS Policlinico San Matteo, Pavia, Italy; 4 Department of Medical and Surgical Sciences (DIMEC), University of Bologna, Bologna, Italy; 5 Dipartimento Oncologia − Comprehensive Cancer Center, Fondazione IRCCS Policlinico San Matteo, Pavia, Italy; 6 Department of Internal Medicine and Medical Therapy, University of Pavia, Pavia, Italy

**Keywords:** epithelial-to-mesenchymal transition (EMT), ICI resistance, IFN-γ, immune checkpoint inhibitor (ICI), Non-Small Cell Lung Cancer (NSCLC), PD-L1, tumor plasticity, tumor stemness

## Abstract

Immune checkpoint inhibitors (ICIs) targeting the PD-1/PD-L1 axis have transformed the treatment of Non-Small Cell Lung Cancer (NSCLC), yet durable responses remain limited to a minority of patients. Tumor plasticity is increasingly recognized as a determinant of immunotherapy resistance, although the tumor-intrinsic contribution of immunomodulatory mediators such as IFN-γ and PD-L1 remains poorly understood. In this review, we examine emerging evidence that IFN-γ and PD-L1, beyond their established roles in tumor-immune interactions, may contribute to the regulation of NSCLC plasticity. IFN-γ can activate non-canonical programs involving dose-dependent ICAM1/PI3K/AKT/Notch1 signaling, BCAT1- and FGF2/β-catenin-mediated metabolic reprogramming, and a putative autocrine TBX21/IL-4 signaling circuit that may sustain stem-like properties. PD-L1, in turn, is functionally integrated into plasticity-associated pathways involving the TGF-β signaling axis, β-catenin stabilization, epithelial-to-mesenchymal transition-associated microRNA networks, YAP/TAZ, CD44 and ATM signaling, as well as TRAF6/BECN1/NF-κB-dependent regulation of autophagy. Collectively, these findings support a role for IFN-γ and PD-L1 as context-dependent modulators of NSCLC plasticity, with potential implications for immunotherapy resistance, and provide a rationale for further exploring combinatorial treatment strategies and patient stratification approaches.

## 1 Introduction

Non-Small Cell Lung Cancer (NSCLC) is the most common histological subtype of cancer affecting the lungs, accounting for 85% of all lung cancers (1). The clinical management of advanced NSCLC has significantly improved with the introduction of targeted therapies such as tyrosine kinase inhibitors (TKIs) (2, 3). Similarly, the introduction of immunotherapy has substantially improved outcomes in NSCLC. The clinical use of immune checkpoint inhibitors (ICIs), targeting PD-1/PD-L1 in NSCLC patients without actionable oncogenic alterations, has indeed led to unprecedented clinical responses (4, 5). Nevertheless, durable clinical benefit remains confined to a minority of patients, with 5-year overall survival rates of approximately 15–23% reported in long-term follow-up studies of ICI monotherapy in advanced NSCLC (4, 6). Resistance to ICIs is linked to the expression of alternative inhibitory immune checkpoints by tumor cells – including TIM-3, LAG-3, TIGIT and VISTA – as well as to the activation of oncogenic pathways and the establishment of an immunosuppressive tumor microenvironment (TME) (7–9). Despite these insights, many aspects of treatment resistance in NSCLC remain poorly understood and require further clarification.

Tumor plasticity has been recognized as a critical factor in recurrence, invasion, and resistance to treatment across various cancer types (10). In NSCLC, tumor stemness and epithelial-to-mesenchymal transition (EMT) have already been linked to acquired resistance to radiation, chemotherapy and targeted therapies (11–14). Consistent evidence also links tumor plasticity to resistance to immunotherapy (15–17). However, the specific tumor cell-intrinsic mechanisms through which immunomodulatory mediators contribute to plasticity-driven resistance remain poorly explored, leaving a significant gap in our understanding of how these factors shape tumor cell states beyond their canonical immunological roles.

In this review, we examine the complex and paradoxical roles played by two key immunological factors − the cytokine IFN-γ and the immune checkpoint PD-L1 − in shaping NSCLC plasticity, with particular emphasis on their influence on cellular states and behavior. Notably, available studies have largely investigated IFN-γ- and PD-L1-associated pathways separately, indicating that they may influence tumor plasticity through distinct mechanisms. Nevertheless, these pathways may also intersect and potentially converge on shared molecular programs in selected contexts. In particular, the well-established induction of PD-L1 by IFN-γ provides a plausible point of crosstalk between them (18), although whether this molecular connection translates into functional convergence in the regulation of tumor plasticity has not yet been directly demonstrated.

By examining these interactions, we aim to provide insights into how IFN-γ and PD-L1 may contribute to ICI response and resistance, as well as their broader implications for cancer treatment strategies.

## 2 Tumor plasticity, EMT and stemness: a blurred line

In the intricate landscape of tumor survival mechanisms, EMT and stemness are closely intertwined concepts that contribute to tumor plasticity and, consequently, to tumor progression, metastasis and therapy resistance.

Tumor plasticity refers to the ability of cancer cells to dynamically shift between diverse cellular states in response to microenvironmental cues or therapeutic pressure, thereby acquiring adaptive advantages that promote survival under adverse conditions (19). These changes may arise through genetic, transcriptional, and epigenetic mechanisms, and confer significant advantages on tumor cells in terms of invasion, metastasis and drug resistance, establishing cellular plasticity as a well-recognized hallmark of cancer (20). Accordingly, tumor cells should not be viewed as static entities, but rather as dynamic systems that continuously adapt to ever-changing environmental pressures.

Currently, processes and phenotypic programs such as tumor stemness, EMT, transdifferentiation and metabolic or epigenetic reprogramming – historically viewed as separate biological processes – are recognized as interconnected mechanisms collectively shaping tumor plasticity (20–23). Of note, drug-tolerant persister cells and cellular senescence have also recently been implicated in tumor plasticity, representing distinct, non-genetic cell states that can promote stemness-like features and, in the case of senescent cells, remodel the surrounding microenvironment via their secretory phenotype (SASP) (24, 25).

Among these processes, EMT is the most widely documented manifestation of tumor plasticity, enabling epithelial tumor cells to acquire mesenchymal-associated traits linked to increased motility, invasiveness and therapeutic resistance (16, 19). Rather than a binary transition between epithelial and mesenchymal states, EMT is increasingly viewed as a dynamic and reversible spectrum of cell states, including hybrid epithelial/mesenchymal phenotypes (16). Importantly, EMT and stemness are closely interconnected, as EMT-associated cell-state transitions can promote the acquisition of stem-like properties (16, 26).

Within this framework, tumor stemness refers to the acquisition of stem cell-like properties by cancer cells, including self-renewal, pluripotency, tumorigenic potential and therapy resistance (27). The identification of cancer stem cells (CSCs) as a distinct tumor subpopulation, first demonstrated by Bonnet and Dick in hematologic malignancies, initially suggested a static hierarchical model of tumor organization (28). However, this classical cancer stem cell theory (CSCT) has been progressively challenged by evidence showing that stem-like properties are not restricted to a fixed subpopulation, but can dynamically emerge within tumors in response to microenvironmental and therapeutic stimuli, as proposed by the stemness phenotype model (SPM) (29, 30). Cancer stemness is now recognized as a form of plasticity itself, as demonstrated by the ability of tumor cells to dynamically shift between non-stem cell and stem cell states (10, 31). This conceptual convergence is mirrored at the molecular level, as features traditionally associated with cellular stemness and EMT frequently overlap across multiple tumor types (31, 32). Accordingly, **Table 1** provides an overview of the main markers associated with EMT and/or tumor stemness in NSCLC.

**Table 1:** Markers of EMT and stemness in NSCLC: structure, functional roles, and associated processes.

Marker	Structure	Functions	Associated processes	Ref
ABCG2	Membrane-bound ABC transporter protein (~72 kDa)	Drug efflux; xenobiotic transport; cellular detoxification	Stemness	(33–35)
ALDH1	Polypeptide tetramer (50−55 kDa)	Alcohol metabolism; oxidative stress response; evasion of apoptosis; regulation of Wnt/β-catenin; Notch and Hedgehog pathways	Stemness/EMT	(36–38)
BMI-1 (RNF51, PCGF4)	Polycomb repressive complex 1 component (~37 kDa)	Epigenetic self-renewal regulation; p16INK4a/p19ARF repression; EMT induction; apoptosis suppression; drug resistance	Stemness/EMT	(38–40)
β-catenin	Transcriptional coactivator/adherens junction component (~92 kDa)	Wnt effector; cell adhesion; EMT induction; stemness maintenance; drug resistance via Notch3-β-catenin axis	Stemness/EMT	(41–43)
CD44	Type I transmembrane glycoprotein (85−250 kDa)	Hyaluronic acid receptor; cell adhesion and migration; TME interaction; immune evasion; regulation of Wnt/β-catenin, Hedgehog and PI3K/AKT pathways	Stemness/EMT	(44–47)
CD90 (Thy-1)	Glycosylphosphatidylinositol-anchored glycoprotein (25−35 kDa)	Cell adhesion; immune modulation; ECM interaction; Wnt/β-catenin, Notch and TGF-β pathways	Stemness/EMT	(48–50)
CD117 (c-kit, SCFR)	Type I transmembrane glycoprotein (~145 kDa)	Receptor tyrosine kinase; PI3K/AKT, MAPK/ERK, JAK/STAT pathways; hematopoiesis; angiogenesis	Stemness	(51–53)
CD133 (Prominin-1)	Cholesterol-binding five-fold transmembrane glycoprotein (97−120 kDa)	Angiogenesis; VEGF interaction; Wnt/β-catenin and Notch signaling	Stemness	(38, 45, 54, 55)
CD166 (ALCAM)	Type I transmembrane glycoprotein (100−105 kDa)	Cell adhesion and migration; TME interaction; Wnt/β-catenin, Notch and PI3K/AKT pathways	Stemness	(45, 56, 57)
Collagen type I	Triple-helix fibrous protein (~300 kDa)	Structural ECM support; tissue integrity; wound healing; cell adhesion; angiogenesis	EMT	(58–61)
CXCR4	G-protein coupled receptor (40−70 kDa)	Chemokine receptor; cell migration; immune cell trafficking; bone marrow niche regulation	Stemness/EMT	(62–65)
E-cadherin (CDH1)	Type I transmembrane glycoprotein (~120 kDa)	Epithelial cell adhesion; cell polarity; EMT suppressor; loss drives invasion via EGFR-MEK/ERK/ZEB1/MMP2 axis	EMT	(66–69)
EpCAM	Single transmembrane protein (30−40 kDa)	Cell adhesion; proliferation; EMT; angiogenesis; Wnt/β-catenin, Notch and MAPK/ERK pathways	Stemness/EMT	(45, 57, 70)
Fibronectin (FN1)	Multi-domain ECM glycoprotein (220−250 kDa)	Cell adhesion; tissue remodelling; angiogenesis; integrin, FAK, PI3K/AKT, Notch pathways	EMT	(71–73)
FZD	G-protein coupled receptor (~64 kDa)	Wnt signaling receptor; cell migration; angiogenesis; tissue repair	Stemness/EMT	(74–76)
Integrins (α9β1, α5β1, αvβ6)	Type I transmembrane glycoproteins (185−220 kDa)	ECM adhesion; cell migration; tissue remodeling; Wnt/β-catenin, FAK, PI3K/AKT and MAPK pathways	EMT	(77–79)
LGR5	G-protein coupled receptor (~100–105 kDa)	Wnt/β-catenin receptor; self-renewal; tumor progression; metastasis	Stemness	(80–82)
MMP-2, MMP-9, MMP-19	Metalloproteinases (28−92 kDa)	Tissue remodeling; cell migration; metastasis; angiogenesis	EMT	(83–87)
N-cadherin (CDH2)	Type I transmembrane glycoprotein (130−140 kDa)	Cell adhesion; EMT; Wnt/β-catenin, PI3K/AKT and MAPK pathways	EMT	(66, 69, 88)
Notch1/Notch3	Type I transmembrane receptors (~300 kDa)	CSC maintenance; EMT induction; EGFR-TKI resistance via Notch3-β-catenin axis; stemness maintenance	Stemness/EMT	(89–92)
Slug (SNAI2)	Zinc finger transcription factor (~30 kDa)	EMT induction; apoptosis resistance; stemness regulation; DNA damage response	Stemness/EMT	(93–95)
Snail (SNAI1)	Zinc finger transcription factor (~29 kDa)	EMT induction; E-cadherin repression; invasion; therapy resistance; Wnt/β-catenin and TGF-β pathways	Stemness/EMT	(94, 96–99)
SOX2, Oct4, Nanog	Transcription factors(~34–39 kDa)	Pluripotency network regulation; self-renewal; EMT induction	Stemness/EMT	(13, 100)
SP (Side-population cells)	N/A	Hoechst 33342 dye efflux via ABC transporters	Stemness	(101–103)
Twist1	Basic Helix-Loop-Helix transcription factor (~21 kDa)	EMT induction; metastasis; angiogenesis; p53-mediated apoptosis suppression	EMT	(104–107)
Vimentin	Type III intermediate filament protein (~53 kDa)	Cytoskeleton; cell shape; intracellular transport; EMT; PI3K/AKT and MAPK pathways	EMT	(108–110)
YAP/TAZ	Transcriptional coactivators, Hippo pathway effectors(~44–54 kDa)	EMT and stemness regulation; mechanotransduction; PD-L1 transcriptional regulation; immune evasion; drug resistance	Stemness/EMT	(111–115)
ZEB1/2	Zinc finger/homeodomain transcription factors(~124–136 kDa)	EMT induction; stemness maintenance; therapy resistance; tumor invasion, chemoresistance, miR-200 family repression	Stemness/EMT	(116–120)

NSCLC, Non-Small Cell Lung Cancer; ABC, ATP-binding cassette; VEGF, vascular endothelial growth factor; ALDH, aldehyde dehydrogenase; IL-1, interleukin-1; TME, tumor microenvironment; ECM, extracellular matrix; TGF-β, transforming growth factor beta; SCFR, stem cell factor receptor; LGR5, leucine-rich G repeat-containing protein-coupled receptor 5; BMI-1, B-cell-specific Moloney murine leukemia virus integration region 1; EpCAM, epithelial cell adhesion molecule; EMT, epithelial-to-mesenchymal transition; ABCG2, ATP-binding cassette sub-family G member 2; CXCR4, C-X-C chemokine receptor type 4; FZD, Frizzled receptors; N/A, not applicable.

It is now widely accepted that the plastic loss of lung epithelial lineage identity through dedifferentiation, together with the acquisition of stem cell-like properties, contributes to tumor progression and aggressiveness and is associated with poorer prognosis (121–123).

Given this direct link between plasticity-driven phenotypes and clinical outcomes, identifying the mechanisms underlying tumor plasticity and its role in mediating therapeutic resistance is essential to prevent tumor adaptations that promote treatment failure. In this context, increasing evidence suggests that IFN-γ and PD-L1 may play a previously underappreciated role in shaping tumor plasticity programs in NSCLC.

## 3 Unveiling the role of IFN-γ in shaping tumor plasticity

### 3.1 IFN-γ: core functions and canonical pathways in tumor cells

In the field of tumor immunology, IFN-γ is widely recognized as a highly pleiotropic cytokine with paradoxical, context-dependent effects. Primarily produced by T lymphocytes and natural killer (NK) cells, it exhibits opposing roles, exerting both antitumor and protumor effects (124). IFN-γ gene expression signatures, derived from bulk and single-cell transcriptomic data, were developed to estimate IFN-γ pathway activity across multiple tumor types and assess its association with prognosis and immunotherapy response (125–128). Although not yet translated into routine clinical use, these approaches underscore the biological relevance of IFN-γ signaling heterogeneity in cancer and its dual role.

While the immunomodulatory effects of this cytokine in the TME have been extensively reviewed (129–131), we focus specifically on the paradoxical impact of this cytokine on tumor cells.

The well-established cytotoxic and cytostatic effects of IFN-γ on tumor cells position it as one of the key antitumoral cytokines, particularly in the context of ICI-mediated reactivation of cytotoxic T cells (131). **Figure 1** depicts the main pathways and mechanisms through which IFN-γ exerts its protumor and antitumor effects on cancer cells. Upon binding to its receptor (IFNGR), IFN-γ activates its canonical JAK/STAT signaling pathway, which – through the induction of IFN-γ-responsive genes, including IRF1 – triggers multiple growth-inhibitory and cytotoxic programs in tumor cells. Among these, one of the best-characterized is its cytostatic activity, mediated by the downregulation of cyclins and cyclin-dependent kinases (CDKs), and the upregulation of cell cycle inhibitors, ultimately leading to cell cycle arrest and cellular senescence (132–135) (**Figure 1A**). IFN-γ-mediated reprogramming of tumor cell metabolism has also been reported, further contributing to the suppression of tumor growth (136) (**Figure 1B**). Beyond these cytostatic mechanisms, IFN-γ can also promote autophagy, which can contribute to both tumor cell growth inhibition and death (137, 138) (**Figure 1C**). Moreover, IFN-γ can exert cytotoxic and cytostatic effects on tumor cells through the induction of nitric oxide synthase (NOS2), resulting in increased local nitric oxide (NO) levels, nitrosative stress and ultimately cell cycle arrest and apoptosis (136) (**Figure 1D**). In addition, IFN-γ-responsive gene transcription also induces apoptosis-associated mediators, including caspases and TRAIL (**Figure 1E**). This cascade ultimately induces tumor cell death, further underscoring the essential role of IFN-γ in immune-mediated tumor elimination (131, 139–144). Beyond apoptosis, IFN-γ can also promote ferroptosis, a form of iron-dependent cell death driven by lipid peroxidation (145) (**Figure 1F**). In addition to the mechanisms described above, IFN-γ also enhances tumor immunogenicity through the upregulation of MHC molecules on the tumor cell surface, thereby promoting antigen presentation and immune recognition (131, 146) (**Figure 1G**). Paradoxically, the same IFN-γ-driven upregulation of MHC-I can reduce tumor cell susceptibility to NK cell-mediated killing by promoting the engagement of inhibitory NK-cell receptors (147) (**Figure 1G**). Indeed, despite its well-established antitumor activity, IFN-γ can also facilitate tumor adaptation and progression. Canonical IFN-γ signaling also induces PD-L1 and IDO1 expression in tumor cells, thereby inhibiting T-cell activation and promoting tryptophan depletion and local immunosuppression (146, 148) (**Figures 1H, I**). Moreover, IFN-γ can promote the secretion of pro-angiogenic chemokines such as IL-8/CXCL8 by tumor cells, thereby stimulating tumor angiogenesis (149) (**Figure 1J**).

**Figure 1:**
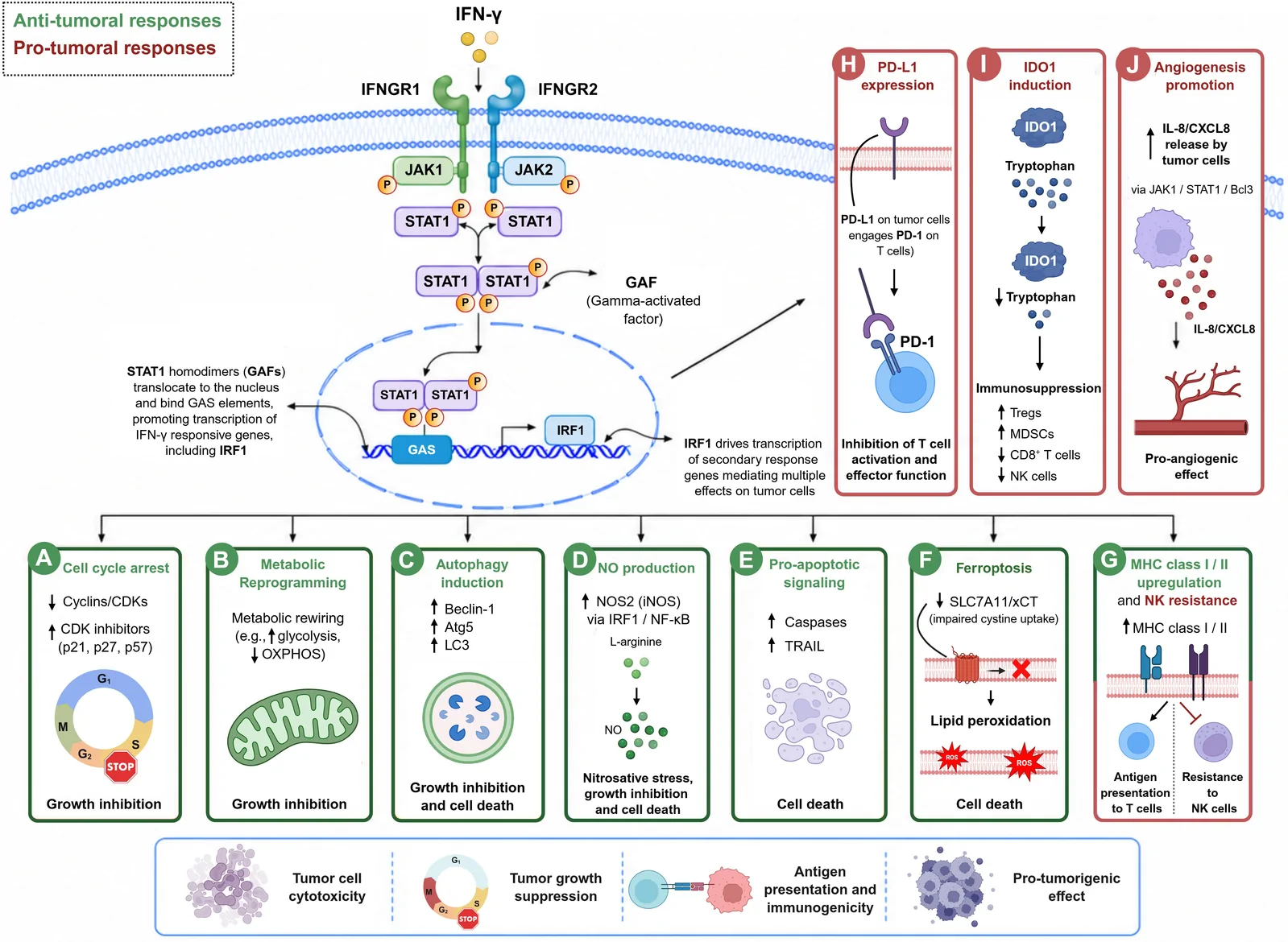
Canonical IFN-γ signaling pathways in tumor cells. Following IFN-γ binding to its receptor complex IFNGR1/2, JAK1 and JAK2 transphosphorylate and activate STAT1 homodimers, known as gamma-activated factors (GAFs), which translocate in the nucleus and bind gamma-activated sequence (GAS) elements, promoting the transcription of IFN-γ-responsive genes, including IRF1. IRF1, together with other STAT1-induced transcriptional regulators, in turn drives the transcription of a broad set of secondary response genes mediating multiple effects on tumor cells: **(A)** cell cycle arrest and dormancy, via downregulation of cyclins/CDKs and upregulation of CDK inhibitors; **(B)** metabolic reprogramming toward increased glycolysis and reduced oxidative phosphorylation (OXPHOS), associated with reduced tumor growth; **(C)** autophagy induction, via IRF1-dependent upregulation of autophagosome formation proteins (Beclin-1, Atg5, LC3); **(D)** nitric oxide (NO) production, via IRF1/NF-κB-mediated transcriptional induction of NOS2, which synthesizes NO from L-arginine, exerting cytotoxic and cytostatic effects through nitrosative stress; **(E)** pro-apoptotic signaling, via upregulation of caspases and TRAIL; **(F)** ferroptosis induction, via transcriptional downregulation of the cystine–glutamate antiporter subunit SLC7A11/xCT, impairing cystine uptake and promoting lipid peroxidation; **(G)** modulation of tumor immunogenicity, through upregulation of MHC class I and II molecules on the tumor cell surface, resulting in enhanced antigen presentation to T cells and increased resistance to NK-cell-mediated killing; **(H)** PD-L1 upregulation (via JAK/STAT1 and PI3K-AKT crosstalk) and **(I)** IDO1 induction, which depletes local tryptophan, suppresses cytotoxic T-cell and NK cell activity, and expands Tregs and MDSCs within the TME; **(J)** angiogenesis stimulation, via release of IL-8/CXCL8 by tumor cells, through the JAK1/STAT1/Bcl3 signaling axis. IFNGR, IFN-γ receptor; CDKs, cyclin-dependent kinases; NOS2, nitric oxide synthase 2; iNOS, inducible nitric oxide synthase; NO, nitric oxide; MHC, major histocompatibility complex; MDSCs, myeloid-derived suppressor cells; TME, tumor microenvironment.

Beyond the canonical mechanisms described above, IFN-γ is increasingly emerging as a mediator of tumor cell plasticity, affecting the adaptability of tumor cells to therapeutic pressures and changes in the microenvironment.

### 3.2 IFN-γ modulation of NSCLC plasticity: preclinical and clinical evidence

Recent studies have shown that IFN-γ can play a key role in modulating cellular regeneration and plasticity in non-tumor contexts. For instance, in response to intestinal epithelial damage, IFN-γ has been shown to mediate a distinctive differentiation process in crypt base-associated stem and progenitor cells, leading to the proliferation of a hybrid colonocyte lineage that, although terminally differentiated, retains fetal-like regenerative and plastic capacities (150). Notably, although mechanistically distinct, this observation is consistent with *in vivo* evidence that sustained IFN-γ exposure can profoundly remodel the intestinal mucosa (151). Similarly, IFN-γ has been reported to enhance the plasticity and immunomodulatory activity of mesenchymal stem cells (MSCs) by modulating their differentiation, proliferation, and self-renewal capacities (152, 153).

The paradoxical role of IFN-γ in tumor growth has been widely discussed in the literature, with studies showing that IFN-γ can support tumor progression by inducing immunosuppressive pathways and reshaping the TME (124, 131, 154). Several studies have also demonstrated that IFN-γ can induce EMT across different tumor types, including lung cancer (155–157).

Overall, available evidence suggests that IFN-γ may induce stem-like phenotypes in NSCLC and other preclinical lung cancer models through three main, non-mutually exclusive mechanisms: dose-dependent activation of non-canonical signaling pathways, metabolic reprogramming of tumor cells and establishment of autocrine signaling circuits (**Figure 2**). Of note, additional processes, including modulation of inflammation and interactions with the TME, may further shape these responses.

**Figure 2:**
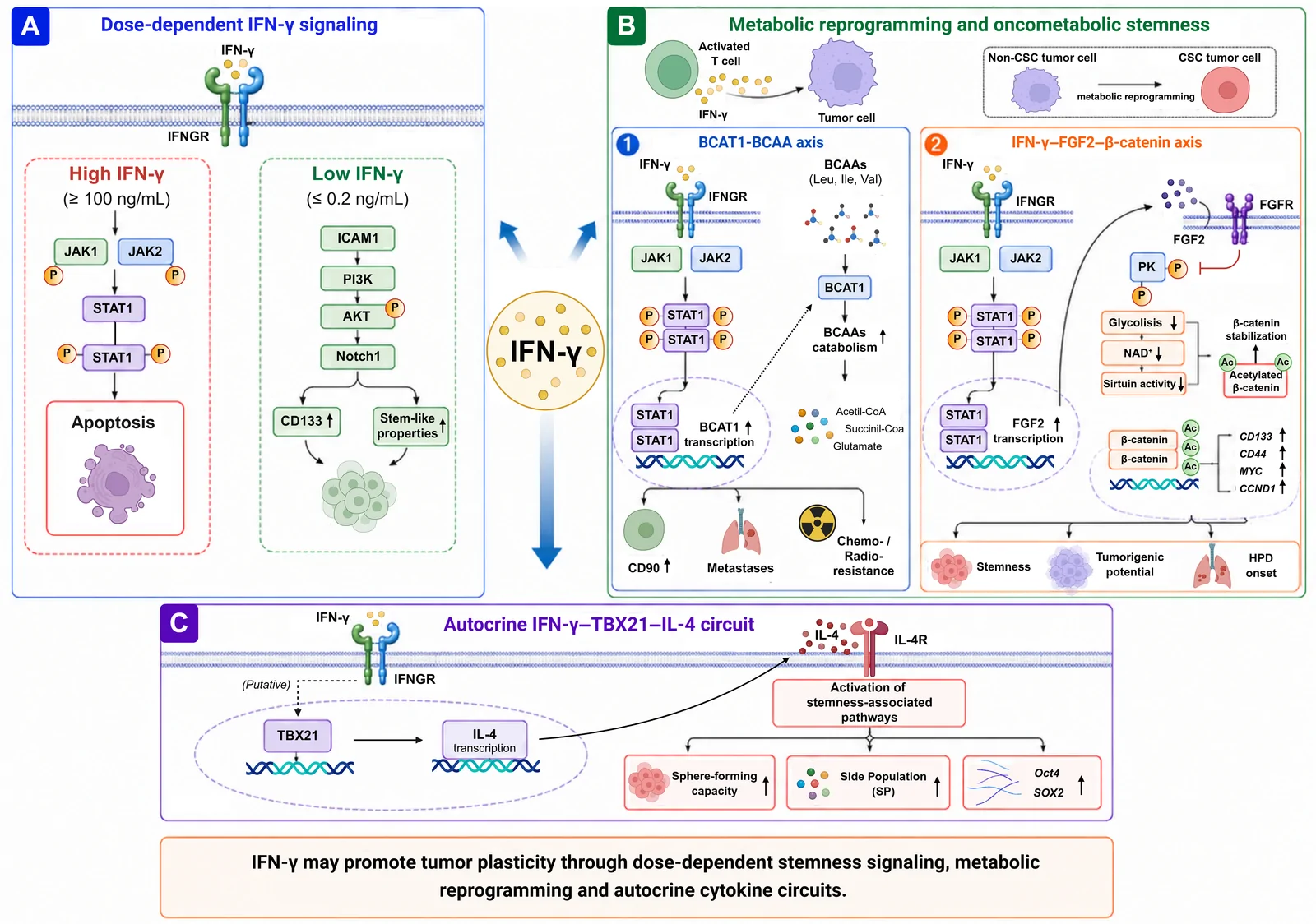
IFN-γ-driven non-canonical mechanisms regulating tumor plasticity in NSCLC. Schematic representation of the concentration-dependent signaling, metabolic reprogramming and autocrine circuits through which IFN-γ may promote stem-like features in NSCLC cells. **(A)** Dose-dependent effects of IFN-γ signaling. High IFN-γ concentrations (≥ 100 ng/mL) predominantly activate the canonical JAK–STAT1 pathway, resulting in tumor-cell apoptosis. Conversely, low IFN-γ concentrations (≤ 0.2 ng/mL) activate the ICAM1/PI3K/AKT/Notch1 signaling cascade, leading to increased CD133 expression and acquisition of stem-like properties. **(B)** IFN-γ-mediated metabolic reprogramming. IFN-γ released by activated T cells promotes the conversion of non-CSC tumor cells into CSC-like cells through two interconnected metabolic mechanisms: (1) in the BCAT1–BCAA axis, IFN-γ-dependent JAK–STAT1 signaling induces BCAT1 transcription, enhancing branched-chain amino acid catabolism and promoting CD90 expression, lung metastatic capacity and resistance to chemotherapy and radiotherapy; (2) in the IFN-γ–FGF2–β-catenin axis, IFN-γ induces FGF2 transcription and extracellular secretion. Autocrine activation of FGFR promotes phosphorylation and inactivation of pyruvate kinase, impairing glycolysis, decreasing intracellular NAD^+^ levels and reducing NAD^+^-dependent sirtuin deacetylase activity. The resulting increase in β-catenin acetylation enhances its nuclear translocation and transcriptional activity, inducing stemness- and tumorigenicity-associated genes, including *CD133, CD44, MYC* and *CCND1*, and ultimately promoting stemness, tumorigenic potential and hyperprogressive disease development. **(C)** Proposed autocrine IFN-γ–TBX21–IL-4 stemness circuit. A plausible, yet unverified, IFN-γ–TBX21 link may initiate the circuit, potentially inducing TBX21 in tumor cells. The dashed arrow denotes the putative interaction between IFN-γ and TBX21, which has not yet been validated in tumor cells. TBX21 promotes IL-4 transcription in tumor cells. Secreted IL-4 subsequently binds IL-4R on the tumor-cell surface, activating autocrine stemness-associated pathways that increase sphere-forming capacity, expand the SP fraction and upregulate the stemness markers Oct4 and SOX2. CSC, cancer stem cells; BCAT1, branched-chain amino acid aminotransferase 1; BCAAs, branched-chain amino acids.

#### 3.2.1 Dose-dependent IFN-γ signaling and stemness induction

A major determinant of the plastic response to IFN-γ is cytokine concentration, which can activate NSCLC cells toward distinct, and even opposing, signaling programs (**Figure 2A**). Song and colleagues demonstrated how prolonged exposure (6 days) to low and high concentrations of IFN-γ activated distinct pathways in human NSCLC cells cultured under 2D and 3D conditions, resulting in markedly different outcomes (142). Notably, while the physiological relevance of the high-dose condition is less clear, the low-dose condition used throughout the study was supported by measurements in tumor interstitial fluid from fresh NSCLC specimens (142). In this study, high IFN-γ concentrations (≥ 100 ng/mL) activated the canonical JAK/STAT1 pathway, leading to tumor cell apoptosis, whereas low IFN-γ concentrations (≤ 0.2 ng/mL) induced the expression of stemness and mesenchymal markers, including CD133, vimentin, fibronectin, ZEB2, Snail and Slug, and enhanced stem-like properties through activation of the ICAM1/PI3K/AKT/Notch1 signaling cascade. This mechanism was further validated *in vivo* in three distinct NSCLC xenograft models generated by subcutaneous or intravenous injection of human NSCLC cells into immunodeficient mice (142). Across these models, low-dose IFN-γ consistently enhanced tumor growth and metastatic dissemination in an ICAM1-dependent manner. Further supporting the clinical relevance of this axis, analyses of human NSCLC specimens and The Cancer Genome Atlas (TCGA) data showed an association between low IFN-γ levels in the TME or high ICAM1 expression and stem-like and mesenchymal features, including CD133 and vimentin expression, as well as chemoresistance, advanced disease features and poorer clinical outcomes (142). These findings provide clinical correlative support for experimental observations linking ICAM1 expression and low intratumoral IFN-γ levels to stem-like features, chemoresistance, disease progression and adverse outcomes in NSCLC patients (142).

In line with these findings, our recent study provided additional preclinical *in vitro* evidence, showing that prolonged exposure (three weeks) to low-dose IFN-γ (0.1 ng/mL) under 3D culture conditions increased expression of the stemness marker CD44 and enhanced stemness-related features in an ICI-resistant patient-derived NSCLC cell line − a response that, conversely, was not observed at higher cytokine concentrations (158). The concentration-dependent effects of IFN-γ may extend beyond tumor cells to the immune compartment of the TME. In this regard, Zhao and colleagues showed that, in stage I NSCLC, IFN-γ within the TME could polarize tumor-associated macrophages (TAMs) toward an M2 protumor phenotype, thereby promoting tumor growth and immune evasion, but only at concentrations below a specific threshold (200 ng/mL) (159). Conversely, exposure to IFN-γ above this threshold resulted in the downregulation of key M2 macrophage markers, supporting concentration-dependent effects of IFN-γ in both tumor and immune contexts, potentially operating across distinct concentration ranges (159). Of note, this dose-dependent effect of IFN-γ on macrophage polarization was demonstrated in a patient-derived *ex vivo* NSCLC tissue model cultured at the air–liquid interface, which retained native tumor and immune components, highlighting the relevance of this threshold-dependent response in a more physiologically representative setting (159).

Although the studies discussed above provide evidence of concentration-dependent IFN-γ effects in both tumor and immune contexts, several important caveats remain. The IFN-γ concentration thresholds reported in these studies should not be regarded as fixed biological cutoffs, as they are influenced by the specific cellular model, culture conditions, duration of cytokine exposure and experimental readouts. For instance, substantial differences can emerge across studies using the same NSCLC cell line (A549): Song and colleagues identified prolonged exposure to high-dose IFN-γ (≥ 100 ng/mL) as pro-apoptotic (142), whereas Zhao and colleagues, using a different experimental protocol and proliferation-based readout, observed increasing A549 proliferation at concentrations up to 200 ng/mL, followed by a decline at higher concentrations (159). Moreover, IFN-γ doses administered *in vivo* cannot be directly equated with concentrations used *in vitro*, further limiting quantitative comparisons across experimental systems (142). Cross-study comparisons are also complicated by differences in cytokine exposure duration. Translation of this dose-dependent framework to human tumors is further complicated by the spatial heterogeneity of IFN-γ-related immune activity. In human NSCLC, spatial analyses have identified discrete intratumoral immune niches enriched in *IFNG*-expressing cells and interferon-associated programs, suggesting that IFN-γ is unlikely to be uniformly distributed across the TME (160). Similarly, spatial analyses in melanoma have shown that IFN-γ signaling can remain confined to local tumor niches, resulting in heterogeneous cytokine exposure among neighboring tumor cells (161). Accordingly, bulk *IFNG* transcript abundance may not accurately reflect the local concentration or exposure dynamics experienced by individual tumor cells. Further illustrating this limitation, an independent bioinformatics analysis of NSCLC cohorts found that tumors with a high EMT score displayed reduced infiltration of selected activated T-cell populations but, paradoxically, higher *IFNG* mRNA expression (162). These findings therefore caution against extrapolating experimentally defined concentration–response relationships directly to human NSCLC.

Regardless of the exact thresholds, the consistent message across these studies is that IFN-γ can exert concentration-dependent and, in some cases, opposing effects, with the relevant concentration ranges requiring empirical definition within each experimental model.

#### 3.2.2 IFN-γ-driven metabolic reprogramming and oncometabolic signaling

Beyond these concentration-dependent effects, IFN-γ has been reported to promote the conversion of non-CSC tumor cells into CSCs through metabolic reprogramming (**Figure 2B**). Unlike the mechanisms described above, these metabolic effects have not been linked to defined IFN-γ dose ranges and appear instead to depend on active immune engagement after ICI therapy or specific oncogenic contexts, including susceptibility to hyperprogressive disease (HPD).

Lung cancer-specific preclinical support was provided by the murine TC-1 model, in which IFN-γ-treated tumor cells displayed enhanced metastatic potential *in vivo*, while pharmacological inhibition of branched-chain amino acid aminotransferase 1 (BCAT1) significantly attenuated this IFN-γ-induced pro-metastatic effect (163).

The underlying mechanism was more extensively characterized in breast cancer models, where IFN-γ released by activated T cells was shown to induce BCAT1 expression and enhance branched-chain amino acid (BCAA) catabolism, thereby promoting the conversion of non-CSC tumor cells into CSCs (163). This metabolic reprogramming increased CD90 expression, sphere-forming capacity, metastatic potential *in vivo*, and resistance to chemotherapy and radiotherapy (163). In breast cancer models, the IFN-γ/BCAT1 axis was supported by mechanistic evidence both *in vitro* and *in vivo*, as well as by correlative data from ICI-treated patients (163). Thus, although direct validation in human NSCLC is still lacking, the functional evidence obtained in a lung cancer model, together with the more extensive mechanistic characterization in breast cancer, supports consideration of this pathway in the context of NSCLC plasticity. Of note, IFN-γ-associated metabolic reprogramming may also engage oncogenic pathways, suggesting the existence of oncometabolic signaling axes that can promote tumor progression and resistance to immunotherapy. This mechanism was described by Li and colleagues, who showed that T cell-released IFN-γ can stimulate tumor stemness and HPD development by activating an intertwined IFN-γ−FGF2−β-catenin metabolic and oncogenic axis (164). Lung cancer-specific evidence included direct preclinical support from human NSCLC cells and murine lung cancer models, with functional validation *in vivo*, whereas the full molecular cascade was mechanistically dissected primarily in melanoma models. In the mechanistically characterized models, IFN-γ induced the transcription of fibroblast growth factor 2 (FGF2), which, by autocrine activation of FGF receptors (FGFRs) on tumor cells, promoted phosphorylation and inactivation of the glycolytic enzyme pyruvate kinase (PK). This disruption of tumor glycolysis resulted in reduced intracellular NAD^+^ levels, thereby limiting the activity of the NAD^+^-dependent deacetylase Sirtuin and promoting β-catenin acetylation (164) (**Figure 2B**). Increased β-catenin acetylation promoted its nuclear translocation and transcriptional activity. In these models, IFN-γ exposure consequently led to the upregulation of genes that regulate stemness (*e.g.*, *CD133, CD44, MYC*) and tumorigenic potential (*e.g.*, *CCND1*) (164). Notably, this IFN-γ-mediated intrinsic cascade was restricted to a subset of lung cancer and melanoma models prone to developing hyperprogression *in vivo* (164). Corroborating these findings, NSCLC and melanoma patients who developed HPD under ICI therapy exhibited not only increased FGF2 and Wnt/β-catenin gene signatures and pathway activities, but also increased expression of tumor stemness and invasiveness markers compared to responder patients (164). Together, these clinical observations provide correlative support for the preclinical findings and suggest the potential relevance of the IFN-γ–FGF2–β-catenin axis to stemness and ICI-associated hyperprogression in NSCLC.

#### 3.2.3 Autocrine circuits sustaining tumor stemness

In addition to metabolic rewiring, IFN-γ may sustain tumor stemness through autocrine signaling circuits (**Figure 2C**). One proposed mechanism involves TBX21, a transcription factor classically induced by IFN-γ/STAT1 signaling in lymphoid and myeloid cells (165, 166), and highly expressed in lung adenocarcinoma, where its expression is associated with poor prognosis (167). Importantly, direct induction of TBX21 by IFN-γ has not been demonstrated in tumor cells. Therefore, the proposed IFN-γ/STAT1–TBX21 link should be regarded as a putative regulatory circuit rather than an established signaling pathway. Nevertheless, the presence of functional IFN-γ/STAT1 signaling in tumor cells (**Figure 1**), together with the established regulation of TBX21 by this pathway in immune cells, raises the possibility that IFN-γ/STAT1 signaling may similarly converge on TBX21 in the tumor-cell compartment and thereby engage the autocrine TBX21–IL-4/IL-4R circuit in lung adenocarcinoma cells (167). Zhao and colleagues demonstrated that TBX21 can modulate stemness-associated signaling pathways in lung cancer by directly binding to the IL-4 promoter, thereby inducing IL-4 transcription and subsequent secretion (167). Once secreted, IL-4 engages its receptor on cancer cells, activating downstream stemness-related pathways that lead to increased sphere-forming capacity, expansion of the side population (SP) fraction and upregulation of stemness markers such as Oct4 and SOX2 (167). This mechanism was supported by direct preclinical evidence both *in vitro*, using A549 cells, and *in vivo*, in A549-derived xenograft models, where modulation of TBX21 affected tumor growth and stemness-associated features (167). Human correlative support was further provided by lung adenocarcinoma datasets and tumor specimens, showing increased TBX21 expression, an association with poorer prognosis and positive correlations with IL-4 and stemness-related genes (167). Thus, while the upstream IFN-γ–TBX21 connection in tumor cells remains to be experimentally established, the downstream TBX21–IL-4–stemness axis is supported by convergent *in vitro*, *in vivo* and human correlative evidence in lung adenocarcinoma.

More broadly, independent evidence suggests that persistent IFN-γ signaling may contribute to alternative resistance-associated tumor states. In this regard, Memon and colleagues reported that a subset of NSCLC patients with acquired resistance to ICIs retained a persistent and dysregulated IFN-γ-related transcriptional program during treatment, rather than showing complete loss of IFN-γ responsiveness (125). These observations support the possibility that ICI resistance may, in selected contexts, coexist with sustained or aberrantly rewired IFN-γ signaling, although a direct causal contribution of this signaling state to resistance remains to be fully established.

#### 3.2.4 Context-dependent outcomes of IFN-γ and implications for ICI resistance

Despite these emerging pro-plasticity mechanisms, the overall impact of IFN-γ signaling in NSCLC remains difficult to define, as antitumor and pro-tumorigenic outcomes coexist across experimental and clinical settings. In contrast to the mechanisms described above, several studies have also reported a positive association between elevated expression of IFN-γ-related gene signatures in lung tumors and improved clinical outcomes, including disease-free survival (DFS), progression-free survival (PFS), and overall survival (OS) (168, 169). Consistently, defects in the tumor-intrinsic IFN-γ signaling are established mechanisms of resistance to ICI therapy (146, 170, 171). However, the favorable clinical associations observed with endogenous IFN-γ activity have not been recapitulated through exogenous IFN-γ administration. Although outside the NSCLC setting, a randomized phase III trial of adjuvant IFN-γ in patients with high-risk resected cutaneous melanoma failed to show an improvement in either DFS or OS compared with observation, with a numerical trend toward worse outcomes in the IFN-γ arm (172). These discrepancies may have multiple explanations. The association between low intratumoral IFN-γ expression and poor prognosis may partly reflect the previously described pro-tumorigenic effects associated with low-dose IFN-γ exposure, which can promote stemness-related pathways (142). Alternatively, the ability of IFN-γ to induce plastic pathways may occur only in specific patient subsets – such as those prone to developing HPD – which may be characterized by intrinsic alterations in IFN-γ signaling (*e.g.*, JAK2 alterations) or reduced responsiveness to canonical pathways (*e.g.*, IFN-γ-mediated induction of PD-L1) (158, 168). A further, non-mutually exclusive possibility is that an initially inflamed, IFN-γ-responsive tumor phenotype − clinically reflected by elevated IFN-γ-associated gene signatures − may itself evolve toward an adaptive, immunosuppressive resistance program under conditions of chronic IFN-γ exposure. This transition has been experimentally reproduced *in vitro* in glioblastoma cells, where prolonged IFN-γ stimulation drove tumor cells from an inflamed toward an immunosuppressive transcriptional program (173). Consistently, in preclinical melanoma models, sustained IFN-γ signaling was sufficient to promote resistance to ICIs even in the absence of additional genetic alterations (174).

Collectively, these findings highlight the context-dependent duality of IFN-γ signaling in lung cancer, which may be shaped by factors such as cytokine dosage, tumor-intrinsic features, and composition of the immune microenvironment. While elevated IFN-γ signaling has been linked to favorable clinical outcomes in certain clinical settings, accumulating evidence points to a pro-tumorigenic role of low-dose or dysregulated IFN-γ activity in promoting plasticity and resistance to immunotherapy. The key IFN-γ-associated pathways discussed above, together with their molecular triggers and downstream effects on tumor plasticity, are summarized in **Table 2**.

**Table 2:** IFN-γ-associated pathways potentially involved in the regulation of tumor plasticity in NSCLC.

Pathway/axis	Activation trigger	Key molecular effects	Effects on tumor plasticity	Lung cancer–related evidence type	Ref
ICAM1/PI3K/AKT/Notch1 axis	Low-dose IFN-γ (≤ 0.2 ng/mL)	CD133 upregulation; ↑ *KLF4, BMI-1, c-Myc, N-cadherin, vimentin, ZEB2, fibronectin, Snail, Slug*	Stem-like phenotype; ↑ sphere-forming ability; ↑ *in vivo* tumor growth; ↑ metastatic potential	Preclinical: human NSCLC cell lines (A549 and NCI-H460), *in vitro* and *in vivo* (A549 xenograft models); Clinical correlative: patient tissues; TCGA transcriptomic analyses	(142)
BCAT1–BCAA axis	IFN-γ from activated T cells (post-ICI)	BCAT1 transcription; ↑ BCAA catabolism; CD90 upregulation	Stem-like phenotype; ↑ sphere-forming ability (breast cancer cells); ↑ metastatic potential; chemo- and radioresistance (breast cancer cells)	Preclinical: murine lung cancer model (TC-1), *in vivo* (syngeneic, immunocompetent mice)	(163)
FGF2–β-catenin axis	Chronic/context-dependent IFN-γ (HPD-prone models)	PK inactivation → ↓ NAD^+^ → ↓ Sirtuin → ↓ β-catenin deacetylation → ↑ *MYC, CD44, CD133, CCND1*	Stem-like phenotype; ↑ *in vivo* tumor growth; HPD	Preclinical: human NSCLC cell line (A549), *in vitro*; murine lung cancer model (LLC), *in vitro* and *in vivo* (syngeneic, immunocompetent mice); Clinical correlative: patient tissues; transcriptomic analyses	(164)
TBX21–IL-4 autocrine circuit	IFN-γ-associated TBX21 expression (putative)	IL-4 secretion → ↑ IL-4R engagement → ↑ SOX2, Oct4, SP cell fraction	Stem-like phenotype; ↑ sphere-forming ability; ↑ *in vivo* tumor growth	Preclinical: human NSCLC cell line (A549), *in vitro* and *in vivo* (A549 xenograft model); Clinical correlative: patient tissues; transcriptomic analyses	(167)

EMT, epithelial-to-mesenchymal transition; TCGA, The Cancer Genome Atlas; BCAT1, branched-chain amino acid transaminase 1; BCAA, branched-chain amino acids; HPD, hyperprogressive disease; LLC, Lewis lung carcinoma; PK, pyruvate kinase; IL-4R, IL-4 receptor; SP, side population.

Disentangling the mechanisms underlying divergent IFN-γ responses remains a major challenge, calling for further investigation into the molecular and clinical variables that determine whether IFN-γ acts as a tumor suppressor or promoter.

## 4 Unveiling the role of PD-L1 in shaping tumor plasticity

### 4.1 PD-L1: molecular functions and pathways in cancer cells

Programmed Death-Ligand 1 (PD-L1), together with its receptor Programmed Cell Death Protein 1 (PD-1), constitutes one of the major immune checkpoint axes targeted by cancer immunotherapy. Since the discovery in the early 2000s that monoclonal antibody-mediated blockade of the PD-1/PD-L1 interaction enhanced antitumor immunity, therapeutic targeting of PD-L1 has been progressively adopted and integrated into standard-of-care regimens for many cancers (175–178). The efficacy of this therapeutic approach relies on the central immunological role of PD-L1 in maintaining self-tolerance and negatively regulating immune responses, particularly those mediated by T cells, through its interaction with the PD-1 receptor (179, 180). PD-L1 is physiologically expressed by macrophages, dendritic cells (DCs), activated T and B cells, myeloid-derived suppressor cells (MDSCs), and some epithelial cells (181), but its expression can also be co-opted by tumor cells to evade antitumor immune responses (182). In this context, PD-L1-mediated inhibition of activated T cells can facilitate tumor growth (182). Indeed, PD-L1 expression remains the only approved biomarker for selecting NSCLC patients for ICI-based therapies (183, 184). However, its predictive performance is often suboptimal and inconsistent, reflecting the complex and context-dependent biological roles of PD-L1 within the TME (185, 186). The discovery in 2008 that PD-L1 could transduce intracellular signals through its cytoplasmic tail, independently of PD-1 engagement (187), revealed a previously unrecognized role for PD-L1 in promoting tumor cell survival through multiple cell-intrinsic mechanisms. As summarized in **Figure 3**, these signals engage multiple pro-survival and proliferative pathways, including PI3K/AKT, MAPK/ERK and mTOR signaling. Through conserved motifs within its cytoplasmic tail, PD-L1 protects tumor cells from IFN-mediated cytotoxicity and apoptotic stimuli (**Figure 3A**). Mechanistically, this protective effect is associated with attenuation of IFN-induced STAT3 activation and caspase-7-dependent apoptosis (188) (**Figure 3A**). In parallel, PD-L1 stimulates MAPK/ERK signaling, supporting cell cycle progression, migration and invasion (**Figure 3B**), while AKT-dependent activation of mTORC1 and its downstream effectors promotes protein synthesis and tumor cell growth (188, 189) (**Figure 3C**). Beyond these proliferative and pro-survival effects, tumor cell-intrinsic PD-L1 signaling also promotes resistance to DNA damage by attenuating type I IFN responses while sustaining a protective, chronic low-level IFN-β signaling program through the cGAS-STING axis (190) (**Figure 3D**). Moreover, PD-L1 modulates autophagy and the clearance of damaged cellular components (191–193) (**Figure 3E**), and drives metabolic reprogramming toward aerobic glycolysis through AKT/mTOR-dependent upregulation of glucose uptake, further supporting tumor cell fitness and progression (194, 195) (**Figure 3F**).

**Figure 3:**
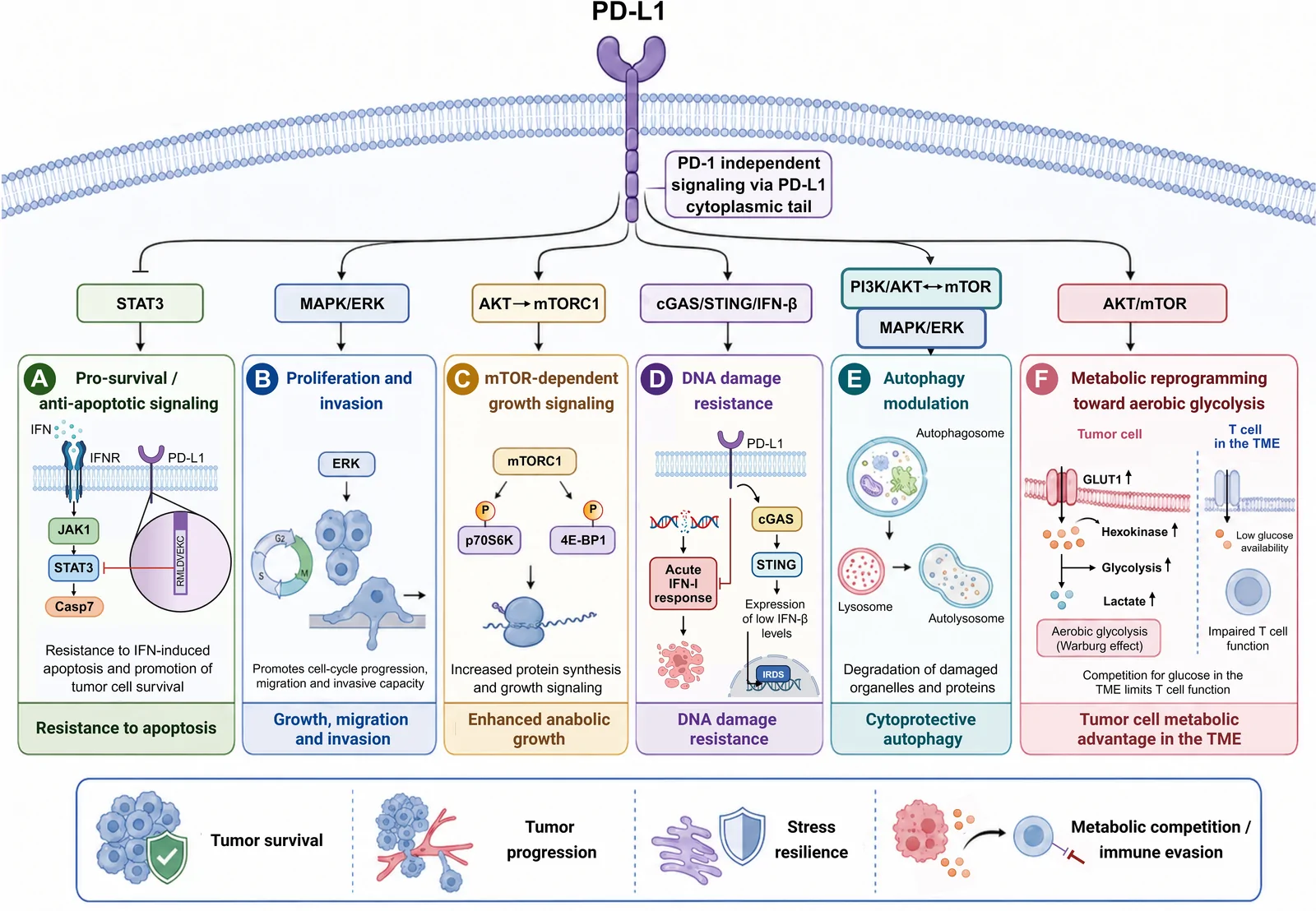
Tumor cell-intrinsic oncogenic signaling pathways driven by PD-L1. PD-L1 transduces intracellular signals independently of PD-1 binding via its cytoplasmic tail, promoting tumor cell survival and progression through multiple mechanisms: **(A)** pro-survival and anti-apoptotic signaling through conserved motifs within the PD-L1 cytoplasmic tail (RMLDVEKC), which attenuate interferon-induced cytotoxicity by limiting STAT3 phosphorylation and CASP7-dependent apoptosis, thereby promoting tumor cell survival; **(B)** proliferation and invasion, via MAPK/ERK pathway activation, promoting cell cycle progression, migration, and invasive capacity; **(C)** mTOR-dependent growth signaling, via AKT-mediated mTORC1 activation and phosphorylation of downstream targets (p70S6K, 4E-BP1); **(D)** DNA damage resistance: PD-L1 suppresses acute cytotoxic IFN-I responses in presence of DNA damage and sustains constitutive cGAS-STING pathway activation, driving low-level, chronic IFN-β production and upregulation of the IFN-related DNA damage resistance signature (IRDS), collectively enhancing tumor cell resistance to radiation and chemotherapy-induced DNA damage; **(E)** bidirectional, context-dependent regulation of autophagy, with PD-L1 either enhancing or suppressing autophagic flux; **(F)** metabolic reprogramming toward aerobic glycolysis, via AKT/mTOR-dependent upregulation of glucose transporters (GLUT1) and hexokinase activity, enhancing the Warburg effect and outcompeting tumor-infiltrating T cells for glucose in the TME. IFNR, IFN receptor; ER, endoplasmic reticulum; TME, tumor microenvironment.

Beyond its oncogenic and immunomodulatory functions, PD-L1 has been implicated in the regulation of cellular plasticity and differentiation across immune cell types, including macrophages, where it modulates lineage differentiation (196), and CD4^+^ T cells, where it promotes Treg polarization (197). These observations point to a broader role of PD-L1 in the regulation of cellular plasticity across different cell types. In line with this, increasing evidence suggests that similar mechanisms may operate in lung cancer, where PD-L1 has been implicated in the modulation of tumor cell plasticity, particularly in NSCLC.

### 4.2 PD-L1 modulation of NSCLC plasticity: preclinical and clinical evidence

Accumulating evidence indicates that PD-L1 expression in tumor cells is generally associated with poorer prognosis in NSCLC, even when positivity is defined using a low cutoff of at least 1% of tumor cells staining positive for PD-L1 (198). Although these observations are complicated by variability in PD-L1 evaluation methodologies across studies, this clinical evidence supports a role for PD-L1 in promoting tumor aggressiveness. Notably, this prognostic association does not appear to be solely attributable to the canonical immunosuppressive function of PD-L1, pointing to additional tumor cell-intrinsic mechanisms operating independently of T cell-mediated immunity (199). Indeed, PD-L1 expression has been associated with an EMT phenotype in lung adenocarcinoma, supporting its link with tumor plasticity (200). Consistently, in the context of NSCLC, converging evidence suggests that PD-L1 may shape tumor plasticity through several interconnected mechanisms, including crosstalk with TGF-β signaling, β-catenin activation, modulation of EMT-related microRNA networks, involvement in stemness-associated pathways and regulation of tumor cell autophagy (**Figure 4**).

**Figure 4:**
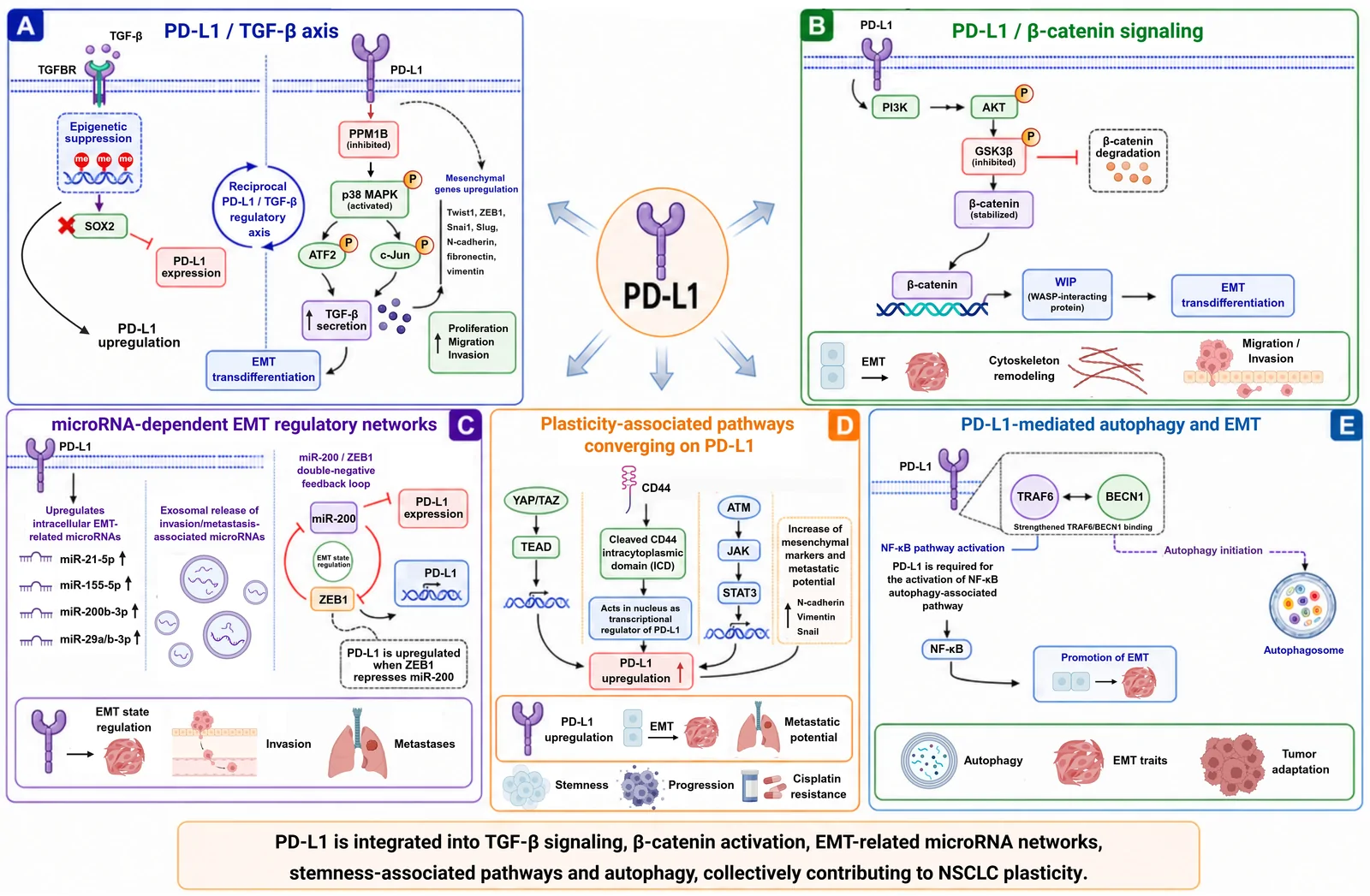
PD-L1-centered regulatory mechanisms associated with tumor plasticity in NSCLC. Schematic representation of tumor cell-intrinsic mechanisms through which PD-L1 may contribute to EMT, stemness-associated phenotypes, metastatic potential and therapy resistance in NSCLC. **(A)** PD-L1 participates in a reciprocal regulatory axis with TGF-β signaling. TGF-β/TGFBR signaling promotes epigenetic suppression of SOX2, relieving SOX2-mediated repression of PD-L1 and leading to PD-L1 upregulation. Increased PD-L1 can further sustain a reciprocal PD-L1/TGF-β regulatory axis through PPM1B inhibition, p38 MAPK activation and ATF2/c-Jun-dependent TGF-β secretion, promoting EMT, mesenchymal gene expression, proliferation, migration and invasion. **(B)** PD-L1 activates the PI3K/AKT pathway, leading to phosphorylation and inhibition of GSK3β. Since active GSK3β normally promotes β-catenin degradation, its inhibition prevents β-catenin turnover, resulting in β-catenin stabilization and downstream transcription of WIP, involved in cytoskeletal remodeling, EMT and enhanced migratory/invasive tumor behavior. **(C)** PD-L1 can occupy both upstream and downstream positions within EMT-related microRNA networks. As an upstream modulator, PD-L1 overexpression induces plasticity-associated intracellular miRNAs and increases the release of exosomal miRNAs linked to invasion and metastasis. Conversely, PD-L1 acts as a downstream target of the ZEB1/miR-200 axis: miR-200 normally represses PD-L1, whereas ZEB1 inhibits miR-200, thereby relieving miR-200-mediated repression and favoring PD-L1 upregulation. This regulatory circuit contributes to EMT state regulation, invasion and metastatic dissemination. **(D)** Multiple plasticity-associated pathways regulate PD-L1 expression at distinct functional levels: YAP/TAZ signaling promotes PD-L1 expression through TEAD-dependent transcriptional regulation, positioning PD-L1 primarily as a downstream target; CD44 contributes through cleavage of its intracellular domain, which can act in the nucleus as a transcriptional regulator of PD-L1, while reciprocal modulation of CD44 and stemness-associated features by PD-L1 supports a bidirectional CD44–PD-L1 circuit; in the context of cisplatin resistance, ATM activates the JAK/STAT3 axis, resulting in PD-L1 induction, with PD-L1 subsequently contributing to the mesenchymal and invasive phenotype. Together, these pathways link PD-L1 upregulation to mesenchymal marker expression, EMT traits, stemness, metastatic potential and cisplatin resistance. **(E)** PD-L1 promotes autophagy-associated EMT through its intracytoplasmic tail, enhancing TRAF6/BECN1 interaction, NF-κB pathway activation and autophagy initiation, thereby supporting EMT traits and tumor adaptation. EMT, epithelial-to-mesenchymal transition.

#### 4.2.1 Reciprocal PD-L1/TGF-β signaling and β-catenin-dependent plasticity

A central mechanism linking PD-L1 to tumor plasticity is its reciprocal crosstalk with TGF-β signaling, which connects PD-L1 expression to EMT-associated cell-state transitions in NSCLC (**Figure 4A**). For instance, cell-based preclinical evidence indicates that TGF-β can directly influence a regulatory axis involving PD-L1 and SOX2 that controls cell-state transitions in both embryonic stem cells (ESCs) and lung adenocarcinoma cells. In their insightful study, Kuo and colleagues demonstrated that SOX2, a key regulator of stemness and self-renewal, can epigenetically repress PD-L1 expression in both ESCs and lung cancer cell lines (201). In this context, SOX2 is associated with an epithelial, stem-like state rather than mesenchymal plasticity, whereas its loss permits a shift toward a more invasive mesenchymal phenotype, as previously described in the context of the epigenetic SOX2/SOX9 switch in lung adenocarcinoma (202). Conversely, TGF-β − well known for its immunosuppressive functions and its ability to promote EMT while antagonizing SOX2-driven epithelial programs (203, 204) − disrupts this SOX2/PD-L1 balance by epigenetically suppressing SOX2 in human NSCLC cell lines, while concomitantly upregulating PD-L1 and inducing EMT transdifferentiation (201, 205). Further supporting the clinical relevance of this regulatory network, correlative analyses of human NSCLC datasets showed an inverse association between SOX2 and TGFBR1/2 expression, with SOX2-low/TGFBR-high tumors displaying poorer survival outcomes (205). Consistently, analysis of primary lung adenocarcinomas from TCGA independently revealed positive correlations between PD-L1 expression and TGF-β receptors (TGFBR1/2) and ligands (TGF-β1, TGF-β2, and TGF-β3) (201). Notably, preclinical evidence from murine melanoma models, both *in vitro* and *in vivo*, has also demonstrated a bidirectional crosstalk between tumor PD-L1 and TGF-β in the regulation of EMT (206). Together, convergent preclinical and clinical correlative data support the existence of a coordinated regulatory loop linking PD-L1 expression, TGF-β signaling and EMT.

In addition to being regulated by TGF-β, PD-L1 has also been implicated in the regulation of EMT-associated pathways that converge on TGF-β signaling. Preclinical *in vitro* studies in NSCLC cell lines showed that PD-L1 overexpression not only upregulated mesenchymal genes – including *Twist1, ZEB1, Snail, Slug, N-cadherin, fibronectin* and *vimentin* – but also promoted TGF-β secretion by directly inhibiting the phosphatase PPM1B, thereby activating the p38 MAPK pathway and its downstream mediators ATF2 and c-Jun (73). These events ultimately enhanced cell proliferation, migration and invasion (73). Interestingly, not only PD-L1 overexpression, but also PD-L1 engagement by recombinant PD-1 increased the expression of EMT-related markers in NSCLC cell lines (73). Moreover, the MEK signaling pathway is also involved in the TGF-β–dependent regulation of PD-L1 expression in ESC differentiation and in primary normal human lung fibroblasts (201, 207), supporting a role for this pathway in the PD-L1–TGF-β axis. The tumor-promoting activity of intrinsic PD-L1 signaling was further supported *in vivo* using PD-L1-overexpressing or -silenced murine lung cancer cells implanted into immunodeficient mice, where PD-L1 enhanced tumor growth, EMT-associated features and metastatic dissemination (73). Human correlative support was further provided by analyses of two ICI-treated NSCLC cohorts, in which a mesenchymal phenotype in PD-L1-high tumors was associated with poorer therapeutic outcomes and a more immunosuppressive TME (73).

In addition to these molecular circuits, preclinical evidence from non-malignant pulmonary fibrosis models indicates that PD-L1 can also modulate fibroblast-to-myofibroblast transition (FMT), a TGF-β-driven form of transdifferentiation (207). Mechanistically, studies in primary human lung fibroblasts showed that TGF-β-induced PD-L1 interacts with Smad3 and promotes GSK3β phosphorylation and β-catenin stabilization, thereby facilitating FMT (207). This mechanism was further supported *in vivo* in murine pulmonary fibrosis models and by increased PD-L1 expression in lung tissues from patients with idiopathic pulmonary fibrosis (207). Given the overlap in signaling pathways that drive tissue remodeling in pulmonary fibrosis and tumor progression in lung cancer (208), these findings suggest that PD-L1/β-catenin signaling may represent a conserved mechanism of cellular plasticity with potential relevance to lung cancer. Accordingly, preclinical studies in NSCLC cell lines demonstrated that PD-L1 activates PI3K/AKT signaling, promoting GSK3β phosphorylation and β-catenin stabilization, which in turn enhances β-catenin–dependent transcription of WASP-interacting protein (WIP) (209) (**Figure 4B**). This pathway was further supported *in vivo* in a human lung cancer xenograft model and by clinical correlative evidence showing a positive association between PD-L1 and WIP expression in lung adenocarcinoma tissues (209). Notably, WIP is a key regulator of actin cytoskeleton reorganization, and its expression has been shown to increase during TGF-β1–induced EMT in the NSCLC cell line A549 (210). Moreover, WIP overexpression has been associated with increased tumor aggressiveness in several cancer types, including prostate, breast and lung cancers (210–212). Consistently, *in vitro* evidence has further supported a tumor-promoting role of PD-L1 in NSCLC cell proliferation, invasion, and aggressiveness through the activation of Wnt/β-catenin and MAPK signaling pathways (213).

Taken together, these findings support interconnected PD-L1-associated TGF-β and β-catenin plasticity pathways, with convergent preclinical evidence from human NSCLC cell lines and *in vivo* lung cancer models, complemented by clinical correlative data from NSCLC datasets, tumor tissues, and patient cohorts, as well as additional mechanistic evidence from melanoma and pulmonary fibrosis models.

#### 4.2.2 Post-transcriptional and plasticity-associated regulatory networks involving PD-L1

Beyond TGF-β- and β-catenin-dependent signaling, PD-L1 is embedded in post-transcriptional and plasticity-associated regulatory programs that can either control its expression or, in selected settings, enable tumor-intrinsic PD-L1 to functionally modulate EMT- or stemness-related phenotypes (**Figures 4C, D**). Importantly, these interactions do not uniformly establish PD-L1 as a causal regulator of plasticity. Rather, they place PD-L1 at different regulatory levels within plastic cell-state programs, acting as an upstream modulator, a downstream target, or a component of reciprocal regulatory circuits. Accordingly, the mechanisms discussed below are distinguished according to the functional position occupied by PD-L1 within each pathway.

These mechanisms include interactions with EMT-regulating microRNAs and signaling pathways involving YAP/TAZ, CD44 and ATM.

In the context of EMT-regulating microRNAs, PD-L1 can occupy distinct regulatory positions. Tumor-intrinsic PD-L1 may act upstream by modulating the expression of plasticity-associated microRNAs or, alternatively, serve as a downstream target of established miRNA-driven EMT programs. Preclinical *in vitro* studies in NSCLC cell lines showed that PD-L1 overexpression altered a broad panel of cellular microRNAs implicated in EMT, invasion and metastasis. Specifically, PD-L1 overexpression increased the expression of several microRNAs involved in the regulation of EMT-related gene expression, including miR-21-5p, miR-155-5p, miR-200b-3p and miR-29a/b-3p, while also altering the abundance of exosomal miRNAs associated with tumor invasion and metastasis, including miR-21-5p, miR-155-5p and members of the miR-29 family (213). These findings suggest that PD-L1 may influence tumor plasticity through the modulation of EMT- and invasion-associated miRNA profiles, although this mechanism is currently supported only by preclinical *in vitro* evidence. Conversely, PD-L1 can also act as a downstream target of microRNA-mediated regulatory programs. For instance, in preclinical NSCLC models, miR-455-5p was shown to directly bind the PD-L1 3′UTR and repress its expression *in vitro*, thereby increasing sensitivity to cisplatin, whereas restoration of PD-L1 rescued the chemoresistant phenotype (214). Human correlative support was provided by analyses of NSCLC lymph-node metastases, primary tumor cohorts, and transcriptomic datasets, in which reduced miR-455-5p expression was associated with increased PD-L1 levels, chemoresistance and alterations in the immune microenvironment (214).

A similar, but more extensively characterized, relationship occurs within the miR-200/ZEB1 regulatory circuit, an established double-negative feedback loop that stabilizes EMT states, in which miR-200 – a suppressor of tumor EMT – downregulates ZEB1 and ZEB1 – a well-known EMT activator –, in turn, transcriptionally represses miR-200 (215). Lung cancer-specific studies identified PD-L1 as a direct downstream target of this circuit: in human NSCLC and murine lung cancer cells, miR-200 overexpression or ZEB1 loss reduced PD-L1 expression, whereas ZEB1 relieved miR-200-mediated repression of PD-L1 (216) (**Figure 4C**).

The functional consequences of this regulation were further demonstrated *in vivo* in syngeneic murine lung adenocarcinoma models, where increased PD-L1 expression promoted T-cell immunosuppression and favored tumor growth and metastatic dissemination (216). Human lung cancer datasets provided additional correlative support for the association among EMT status, the miR-200/ZEB1 circuit and PD-L1 expression (216). Thus, within this circuit, PD-L1 primarily emerges as a downstream consequence of the EMT program.

Beyond microRNA-mediated regulation, PD-L1 is positioned downstream of several plasticity-associated signaling pathways, while also participating in reciprocal regulatory circuits in selected settings (**Figure 4D**). For instance, the activation of TAZ and YAP, major effectors of the Hippo pathway, has been reported to directly upregulate PD-L1 expression in lung cancer cell models through TEAD transcription factors (217). In this setting, PD-L1 therefore acts primarily as a downstream transcriptional target of a plasticity-associated pathway rather than as an established mediator of YAP/TAZ-driven stemness. Similarly, CD44, a well-established marker of tumor plasticity, has been reported to transcriptionally regulate PD-L1 expression in NSCLC through its cleaved intracytoplasmic domain, which can function as an intracellular transcription factor (218). Unlike the YAP/TAZ pathway, however, the CD44–PD-L1 relationship appears to be bidirectional: although PD-L1 initially acts downstream of CD44, PD-L1 depletion or antibody-mediated targeting has also been shown to modulate CD44 expression and alter stemness-related features in a patient-derived NSCLC cell line (158). The CD44–PD-L1 interaction may therefore represent a reciprocal regulatory circuit in which tumor-intrinsic PD-L1 can itself functionally influence stem-like phenotypes. PD-L1 has also been identified as a downstream effector of an EMT- and chemoresistance-associated signaling program driven by the Ser/Thr protein kinase ATM (**Figure 4D**). Under cisplatin treatment and chemoresistant conditions, NSCLC cell lines displayed ATM upregulation, increased expression of mesenchymal markers – including N-cadherin, vimentin and Snail – and enhanced invasive potential (219). Mechanistically, ATM mediated this EMT program by activating the JAK/STAT3 pathway, ultimately resulting in PD-L1 upregulation on tumor cells (219). Importantly, PD-L1 neutralization partially reversed the mesenchymal phenotype and reduced tumor-cell invasion, supporting a functional role for PD-L1 as a downstream mediator, rather than merely a marker, of this ATM-driven EMT program *in vitro* (219). The pro-metastatic role of ATM was further validated *in vivo* in an orthotopic xenograft model, where pharmacological ATM inhibition reduced EMT-associated features and metastatic dissemination (219). Thus, in this setting, PD-L1 acts as a downstream functional mediator of the ATM-driven EMT/chemoresistance program in NSCLC, with its contribution to the mesenchymal and invasive phenotype demonstrated *in vitro*, although its specific role has not yet been validated *in vivo*.

Collectively, these studies position PD-L1 at distinct regulatory levels within plasticity-associated programs, where it may function as an upstream modulator, a downstream target, or, in selected settings, a functional component of reciprocal circuits associated with EMT and stemness. However, mechanistic evidence remains predominantly preclinical, while human correlative support is currently available only for selected regulatory interactions.

#### 4.2.3 PD-L1-dependent autophagy and adaptive plasticity

PD-L1 may also contribute to tumor plasticity by regulating autophagy, an adaptive cellular process increasingly linked to tumor progression, invasion and EMT (193, 220, 221) (**Figure 4E**). Preclinical *in vitro* studies in human NSCLC cell lines showed that tumor-intrinsic PD-L1 promotes Toll-like receptor (TLR)-induced autophagy through the TRAF6/BECN1 signaling axis. Mechanistically, PD-L1 physically associates with TRAF6 (an E3 ubiquitin ligase) and BECN1 (Beclin-1) through its intracellular domains, strengthening the TRAF6–BECN1 interaction, promoting BECN1 ubiquitination, and facilitating autophagy initiation (222). Functional studies showed that PD-L1 depletion impaired TLR-induced autophagy and NF-κB activation, whereas PD-L1 overexpression enhanced both processes (222). Importantly, PD-L1 was required for activation of the NF-κB autophagy-associated pathway, which in turn promoted EMT, thereby functionally linking PD-L1-dependent TRAF6/BECN1-mediated autophagy to EMT regulation in human NSCLC cells (222–224). The tumor-promoting activity of PD-L1 was further supported *in vivo* in an A549 xenograft model, in which PD-L1 knockout reduced tumor growth and metastatic dissemination, although mechanistic validation of the PD-L1/TRAF6/BECN1 autophagy axis remains limited to *in vitro* models (222). Nevertheless, human correlative evidence from transcriptomic analyses of 42 NSCLC tumor specimens showed that coordinated upregulation of *CD274, TLR4, TRAF6*, and *BECN1* was associated with enrichment of autophagy-, invasion-, and metastasis-related gene programs, providing correlative support for the potential relevance of this pathway in NSCLC (222).

Notably, plasticity-related effects associated with PD-L1 modulation have been reported not only following its genetic induction or engagement by PD-1, but also following pharmacological blockade with ICIs or genetic depletion. Indeed, in a patient-derived model, both the genetic depletion of PD-L1 and antibody-mediated blockade resulted in increased CD44 expression, MAPK pathway activation and enhanced *in vitro* growth efficiency (158). Extending these observations *in vivo*, genetic depletion of PD-L1 enhanced the growth of subcutaneous NSCLC xenografts in immunodeficient mice and was accompanied by increased intratumoral AKT and ERK1/2 activation (225). Consistent with this, suppression of PD-L1 expression has similarly been shown to promote tumorigenesis and cancer stem cell-like traits in cholangiocarcinoma preclinical models (226).

These findings suggest that loss or inhibition of PD-L1 may paradoxically favor tumor-promoting phenotypes in specific and still poorly defined cellular contexts. However, the molecular determinants, biological significance and context dependency of these apparent loss-of-function effects remain to be fully elucidated.

Taken together, the available evidence supports a role of PD-L1 in NSCLC tumor cell plasticity, extending beyond its canonical function as an immune checkpoint. Depending on the cellular setting, PD-L1 may participate at different regulatory levels within interconnected signaling, epigenetic, and post-transcriptional programs − including the TGF-β/β-catenin axes, microRNA circuits, stemness-associated pathways and autophagy − thereby promoting EMT, stem-like phenotypes and adaptive cellular processes (**Table 3**). Overall, these findings support a model in which PD-L1 is functionally integrated into plasticity-associated programs in selected contexts, although the direction and biological consequences of these interactions appear to be highly context dependent.

**Table 3:** PD-L1-associated pathways potentially involved in the regulation of tumor plasticity in NSCLC.

Pathway/axis	Activation trigger	Key molecular effects	Effects on tumor plasticity	Lung cancer–related evidence type	Ref
PD-L1/TGF-β axis	TGF-β-mediated SOX2 epigenetic suppression and PD-L1 de-repression	PD-L1–mediated PPM1B inhibition → p38 MAPK activation → ATF2/c-Jun activation → TGF-β secretion → EMT	↑ *Twist1, ZEB1, Snail, Slug, N-cadherin, fibronectin, vimentin*; ↑ cell proliferation and migration; ↑ metastatic potential; ↑ *in vivo* tumor growth	Preclinical: human NSCLC cell lines (A549, H522, H460, H596, CL1-0 and CL1-5), *in vitro*; murine lung cancer model (LLC), *in vitro* and *in vivo* (immunodeficient mice); Clinical correlative: patient tissues; public transcriptomic datasets	(73, 201)
PD-L1–β-catenin axis	PD-L1 intrinsic signaling	PI3K/AKT activation → GSK3β phosphorylation/inhibition → β-catenin stabilization → WIP transcription → ↑ *N-cadherin, vimentin, MMP-9, claudin-1*	↑ cell viability, migration and colony formation ability; ↑ metastatic potential; ↑ *in vivo* tumor growth	Preclinical: human NSCLC cell lines (H1299, A549, H460 and H358), *in vitro* and *in vivo* (A549 xenograft model); Clinical correlative: patient tissues	(209)
PD-L1–microRNA regulatory networks	PD-L1–driven miRNA modulation; EMT-associated ZEB1 induction and miR-200 loss	↑ EMT-associated miRNAs (*e.g.*, miR-29b-3p, miR-21-5p, and miR-125b-5p); ZEB1–mediated repression of miR-200 → PD-L1 de-repression	EMT state stabilization; CD8^+^ T cell suppression; ↑ metastatic potential; ↑ *in vivo* tumor growth	Preclinical: human NSCLC cell lines (H2170, H1299, H157, H1155, H460 and HCC827) *in vitro*; murine lung cancer models (KP-derived and LLC), *in vitro* and *in vivo* (syngeneic, immunocompetent mice); Clinical correlative: patient tissues; public transcriptomic datasets	(213, 216)
Plasticity-associated pathways converging on PD-L1 (YAP/TAZ–TEAD, CD44–PD-L1 and ATM–JAK/STAT3 axes)	Hippo pathway suppression/YAP-TAZ activation; CD44 cleavage; cisplatin-induced ATM signaling	Convergent transcriptional upregulation of PD-L1	EMT; ↑ mesenchymal genes; ↑ cell clonogenicity; ↑ tumorigenicity; ↑ metastatic potential; immune evasion; cisplatin resistance	Preclinical: human NSCLC cell lines (A549, H1299, HCC827, H358, H1915 and H157), *in vitro* or *in vivo* (A549 xenograft model); Clinical correlative: patient tissues; public datasets	(217–219)
TRAF6/BECN1/NF-κB autophagy axis	PD-L1 cytoplasmic tail interactions	Cytoplasmic PD-L1 enhances TRAF6/BECN1 interaction → autophagy initiation and NF-κB activation	EMT; ↑ cell proliferation and migration; ↑ cell clonogenicity; ↑ sphere-forming capacity; ↑ *in vivo* tumor growth; ↑ metastatic potential;	Preclinical: human NSCLC cell lines (A549 and H460), *in vitro* and *in vivo* (A549 xenograft model); Clinical correlative: transcriptomic analyses of patient tissues	(222)

PPM1B, protein phosphatase 1B; EMT, epithelial-to-mesenchymal transition; LLC, Lewis lung carcinoma.

## 5 Concluding remarks

Tumor plasticity is a key adaptive property of cancer cells that enables dynamic transitions between distinct phenotypic states, including EMT and the acquisition of stem-like features, that contribute to tumor progression, immune evasion, and therapy resistance. The acquisition of stem-like states and the activation of EMT programs are recognized mechanisms of treatment resistance and therapeutic failure (227). Collectively, the evidence discussed in this review highlights how IFN-γ and PD-L1, commonly regarded as important players of antitumor immunity, may also participate in transcriptional, metabolic and epigenetic tumor programs, shaping NSCLC plasticity.

In this context, IFN-γ exerts a highly context-dependent influence on tumor behavior, and its effects in NSCLC vary according to cytokine concentration, duration of exposure, cellular model and microenvironmental conditions. Low or dysregulated IFN-γ signaling has been reported to favor tumor plasticity through distinct non-canonical mechanisms. These include the ICAM1/PI3K/AKT/Notch1 axis − supported by direct preclinical and clinical correlative evidence in NSCLC −, metabolic rewiring involving BCAT1 − whose mechanistic characterization derives predominantly from other tumor types but is supported by functional lung cancer models −, and the IFN-γ–FGF2–β-catenin axis, for which lung cancer-specific preclinical and clinical correlative evidence is available. A putative IFN-γ–TBX21–IL-4 circuit may represent an additional mechanism, although the upstream IFN-γ–TBX21 connection in tumor cells remains to be experimentally established (142, 163, 164, 167).

In parallel, in certain settings, PD-L1 may participate in tumor plasticity through several tumor-intrinsic mechanisms, including: reciprocal crosstalk with TGF-β signaling, supported by preclinical NSCLC models and clinical correlative evidence, with complementary findings from melanoma models (73, 201, 205, 206); β-catenin stabilization via GSK3β phosphorylation and WIP upregulation, supported by preclinical NSCLC studies, *in vivo* lung cancer models, correlative analyses of lung adenocarcinoma tissues, and additional mechanistic support from pulmonary fibrosis models (207, 209); modulation of selected EMT- and invasion-associated miRNA networks, for which evidence remains predominantly preclinical (73, 213). Conversely, PD-L1 can also occupy a downstream position within established plasticity-associated programs, as exemplified by the miR-200/ZEB1 circuit, for which preclinical *in vitro* and *in vivo* lung cancer evidence is complemented by human correlative data (216). PD-L1 has also been implicated in autophagy regulation through the TRAF6/BECN1 signaling pathway, supported primarily by preclinical NSCLC evidence and human correlative data, although the specific autophagy axis has not yet been directly validated *in vivo* (222). More broadly, PD-L1 occupies distinct regulatory positions across plasticity-associated pathways: it acts primarily as a downstream target of YAP/TAZ signaling, participates in a reciprocal relationship with CD44, and functions as a downstream mediator of the ATM/JAK/STAT3 EMT program in preclinical *in vitro* models (217–219).

Thus, rather than representing a uniformly causal driver of plasticity, PD-L1 emerges as a context-dependent molecular node whose functional position varies according to the specific regulatory network.

Importantly, the IFN-γ–driven induction of PD-L1 expression provides a plausible mechanistic link between these pathways and raises the possibility that they may converge to support stem-like and EMT-associated tumor states in selected contexts (73) (**Figure 5**). However, direct functional evidence for such convergence in the regulation of tumor plasticity remains limited.

**Figure 5:**
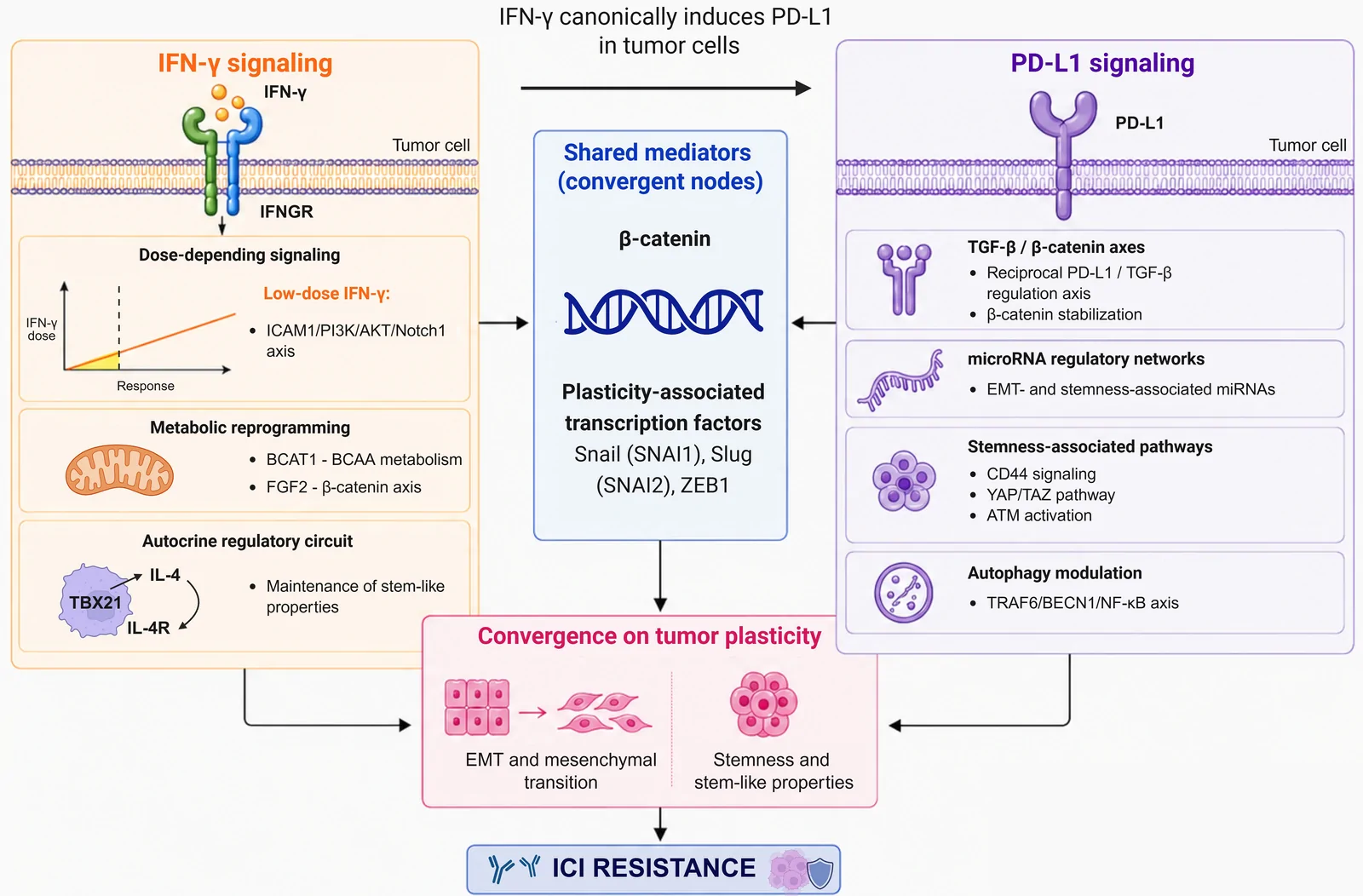
Potential convergence of IFN-γ- and PD-L1-associated pathways on tumor plasticity and ICI resistance in NSCLC. Schematic overview of the major tumor cell-intrinsic mechanisms through which IFN-γ and PD-L1 may shape NSCLC plasticity. Low or chronic IFN-γ signaling may promote EMT and stem-like features through dose-dependent signaling, metabolic reprogramming involving the BCAT1–BCAA and FGF2–β-catenin axes, and the TBX21–IL-4 circuit. In parallel, PD-L1 is integrated into TGF-β/β-catenin signaling, reciprocal microRNA regulatory networks, stemness-associated pathways and autophagy. These pathways may converge on EMT-associated cell-state transitions and stemness, with β-catenin and EMT-related transcription factors representing shared molecular nodes, potentially promoting tumor plasticity and contributing to resistance to ICIs. IFN-γ-mediated induction of PD-L1 further provides a potential link between the two regulatory programs. IFNGR, IFN-γ Receptor; BCAT1, branched-chain amino acid aminotransferase 1; BCAAs, branched-chain amino acids; IL-4R, IL-4 receptor; EMT, epithelial-to-mesenchymal transition; ICI, immune checkpoint inhibitor.

The pro-plasticity effects associated with dysregulated IFN-γ signaling or tumor-intrinsic PD-L1 activity may themselves be restricted to specific cellular contexts characterized by intrinsic tumor features that predispose to ICI resistance or hyperprogression, as suggested by preclinical and correlative studies (164). Given the predominantly preclinical nature of the available evidence, however, the molecular determinants of these tumor states and their clinical relevance in ICI-treated NSCLC remain to be prospectively validated. Defining such determinants may ultimately enable the identification of patients in whom IFN-γ- or PD-L1-associated signaling favors, rather than restrains, tumor progression. Candidate biomarkers emerging from the mechanisms discussed in this review include altered IFN-γ signaling or exposure patterns, activation of FGF2/β-catenin or YAP/TAZ programs, expression of ICAM1, BCAT1, CD44 and other stemness- or EMT-associated markers, as well as composite transcriptional signatures integrating plasticity and immune context. Their predictive value, however, remains hypothetical and requires validation in prospective NSCLC cohorts.

From a therapeutic perspective, these observations raise the possibility that targeting plasticity-associated pathways alongside ICIs could be beneficial in selected resistant tumors. Dual targeting approaches aimed at simultaneously inhibiting complementary EMT-associated pathways are already being explored, including strategies combining PD-L1 blockade with inhibition of TGF-β or β-catenin signaling (228, 229). Other pathways discussed in this review may also represent potential therapeutic targets. Preclinical studies in NSCLC support YAP/TAZ inhibition as a candidate approach, while its combination with PD-1/PD-L1 blockade has been proposed as a means of simultaneously interfering with tumor-intrinsic and immune-evasive programs (114, 230). Autophagy inhibition has also been clinically explored in NSCLC in combination with chemotherapy (231), providing a rationale for investigating whether autophagy targeting could similarly enhance ICI efficacy in selected tumors, particularly in light of the PD-L1/TRAF6/BECN1 axis discussed above. From a metabolic perspective, pharmacological inhibition of BCAT1 has been shown to attenuate IFN-γ-induced metastasis and improve the efficacy of immune checkpoint blockade in preclinical breast and lung cancer models (163). Interestingly, preclinical evidence from NSCLC cell lines also suggests a potential link between BCAT1 and PD-L1 through an epigenetic mechanism: BCAT1-mediated reduction of α-ketoglutarate has been associated with decreased miR-200 expression in lung cancer cells (232). Given that miR-200 can repress PD-L1 expression (**Figure 4C**), these observations raise the possibility that combined targeting of metabolic and epigenetic plasticity − for example, through BCAT1 inhibition together with epigenetic therapies − may warrant investigation in NSCLC. This concept is further supported by the clinical activity of hypomethylating agents and histone deacetylase (HDAC) inhibitors in NSCLC, including reports of durable responses to subsequent anti-PD-1 therapy in a subset of patients (233, 234).

In parallel, single-cell and spatial omics approaches may be particularly valuable for defining putative biomarkers or therapeutic targets and resolving tumor plasticity at higher resolution. Such approaches can identify hybrid cellular states that extend beyond the classical epithelial-to-mesenchymal dichotomy (235), and reconstruct lineage trajectories acquired by tumor cells during treatment, echoing observations from aging-associated mesenchymal drift (236). They may ultimately help determine whether specific plasticity states precede, accompany, or arise as a consequence of ICI resistance, rather than inferring causal relationships from cross-sectional associations alone.

Altogether, the evidence discussed in this review supports a major role for tumor plasticity in immune escape and therapy resistance, with IFN-γ and PD-L1 emerging as context-dependent modulators of this process. Plasticity-associated pathways may therefore represent potential therapeutic vulnerabilities, although their clinical relevance and therapeutic value require further validation in NSCLC.
